# Case report: Tunneling foreign body in the metatarsal bones of a dog

**DOI:** 10.3389/fvets.2022.1039903

**Published:** 2022-11-18

**Authors:** Hannah Wagoner, Merrilee Holland, Katherine Pansini, Jess McCarthy, Kaitlin Fiske

**Affiliations:** ^1^Department of Clinical Sciences, Auburn University, College of Veterinary Medicine, Auburn, AL, United States; ^2^Department of Diagnostic Imaging, Gulf Coast Veterinary Diagnostic Imaging, Houston, TX, United States; ^3^Department of Clinical Sciences, University of Wisconsin-Madison, Madison, WI, United States

**Keywords:** strangulating foreign body, computed tomography, draining tract, dog, lameness, bone lysis

## Abstract

A 2-year-old male intact goldendoodle presented for intermittent lameness, persistent draining tracts, and radiographic identified boney lytic lesions involving the metatarsal region, which had persisted for approximately 8 months before presentation to our referral hospital. Radiographic and computed tomography (CT) images suggested a tubular structure encircling and tunneling through the right metatarsal bones. Exploratory surgery confirmed a circumferential rubber band foreign body, with lytic boney tunneling within the metatarsal bones. CT provided vital information to assist in the surgical planning for this patient with a chronic tunneling foreign body.

## Introduction

Circumferential strangulating foreign bodies, also known as “rubber band syndrome” in humans, is a rare condition that has been documented primarily in small children with linear foreign objects around distal limbs. Rubber band syndrome is characterized by linear external foreign material that encircles distal limbs or cervical anatomy, while progressively constricting blood flow, movement, and damaging musculoskeletal and neurovascular structures. This is mainly reported in India, where ceremonial or religious bands are tied around wrists and ankles and forgotten (1–3, 7, 8). In humans, cases of rubber band syndrome present with pain, edema, non-healing wounds, draining tracts, and decreased function of the limb (1, 2, 7, 8, 12, 16, 18, 19). There is limited literature describing this syndrome in animals, with the majority of reports including cervical foreign bodies in four dogs (4, 6, 13, 17) and one cat (11). The remaining literature describes distal limb foreign bodies or strangulating hair mats in a horse, two dogs, and one cat (5, 9, 10, 14, 15). This study aims to add to the dearth of literature recounting distal limb rubber band syndrome in dogs, and details the presentation, cytologic, and surgical description of distal limb strangulating foreign bodies. Also, to illustrate the CT and radiographic characteristics to improve diagnosis and expedite treatment of rubber band syndrome in small animals.

## Case description

**Figure 1:**
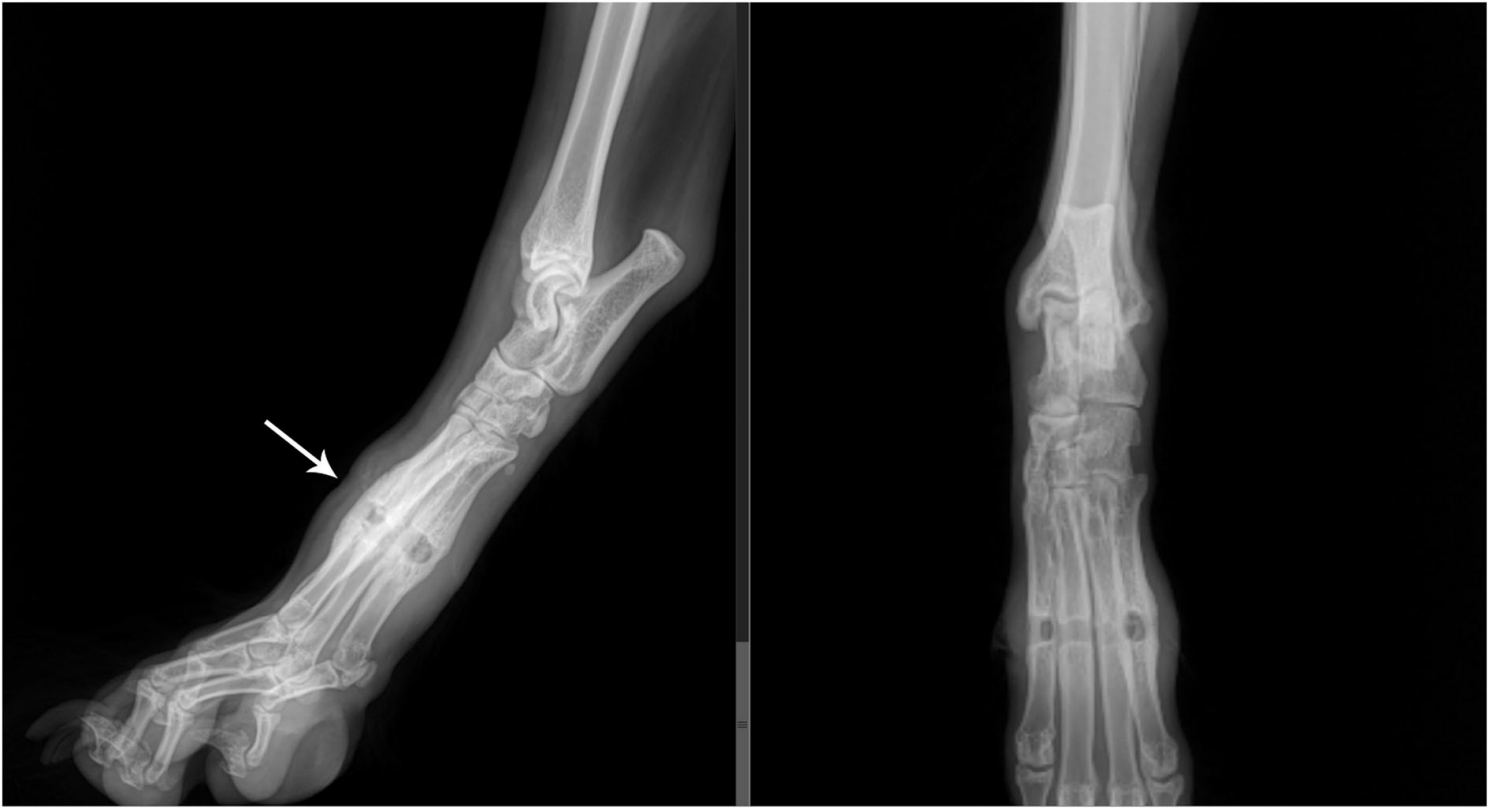
Lateral and dorsoplantar view of the right metatarsus showed mid-diaphyseal, well delineated, horizontal lysis of metatarsals 2–5, with ovoid, discrete geographic lysis of metatarsals 2 and 5. Smoothly marginated new bone is seen on the lateral aspect of metatarsal 5 and the plantar aspect of the metatarsals on the lateral view. Moderate soft tissue swelling was noted surrounding the lucencies with a subtle dorsal indentation of the soft tissues (arrow) at the level of the boney lysis best seen on the lateral view.

A 2.5-year-old male intact goldendoodle presented to Auburn University College of Veterinary Medicine (AUCVM) Orthopedic service for evaluation of a right pelvic limb lameness, persistent draining tracts over the right metatarsals, and associated boney lytic lesions seen on radiographs taken by the referring veterinarian (RDVM). The patient initially presented to the RDVM 8 months earlier for chronic intermittent diarrhea, swelling around the right tarsus, and moderate lameness in the right pelvic limb. The patient was reported to have his right pelvic limb stuck in a kennel door several months prior in the owner's residence, which was believed to be the cause of the lameness. The RDVM noted swelling and purulent draining tracts over the right metatarsus, along with circumferential scarring of the metatarsals and moderate tissue edema cranially and medially. The patient was prescribed cefpodoxime (100 mg, 1.5 tabs PO Q 24 h × 10 days), metronidazole (500 mg PO Q12 h × 5 days), and carprofen (100 mg, ½ tab PO Q 12 h). A week later the patient presented to the RDVM for a recheck. The limping and diarrhea resolved; however, the metatarsal swelling and draining tracts had not. At this time, the RDVM surgically explored the wound for evidence of foreign material, but no reason for the draining tracts was found. A dorsoplantar and lateral radiograph of the right tarsus and metatarsals were taken (Figure 1). The patient was then sent home with clindamycin (300 mg, 1 PO Q 12 h × 14 days) and continuation of the carprofen course ( × 14 days). Five months after the lameness started, repeat radiographs were taken of the right hind, which showed soft tissue swelling with a more pronounced indentation of the soft tissues at the level of the boney lysis. The patient was then referred to AUCVM.

On presentation to AUCVM Orthopedic service, 8 months since the onset of clinical signs, the physical exam findings included a weight of 26.5 kg, a heart rate of 102 beats/min, a panting respiratory rate, and an elevated temperature of 103.5°F (39.7°C). The patient was bright, alert, and responsive. The right popliteal lymph node was enlarged, and swelling over the right metatarsus was noted, with three draining tracts. A black circumferential scar was observed encircling the metatarsals after shaving the limb. The remainder of the physical exam was within normal limits with a resolution of reported lameness on presentation. An orthopedic exam was performed which identified mid-diaphyseal soft tissue swelling and bone thickening of the metatarsal bones. A complete blood count and chemistry were performed and were noted to be unremarkable.

## Diagnostic assessment

**Figure 2:**
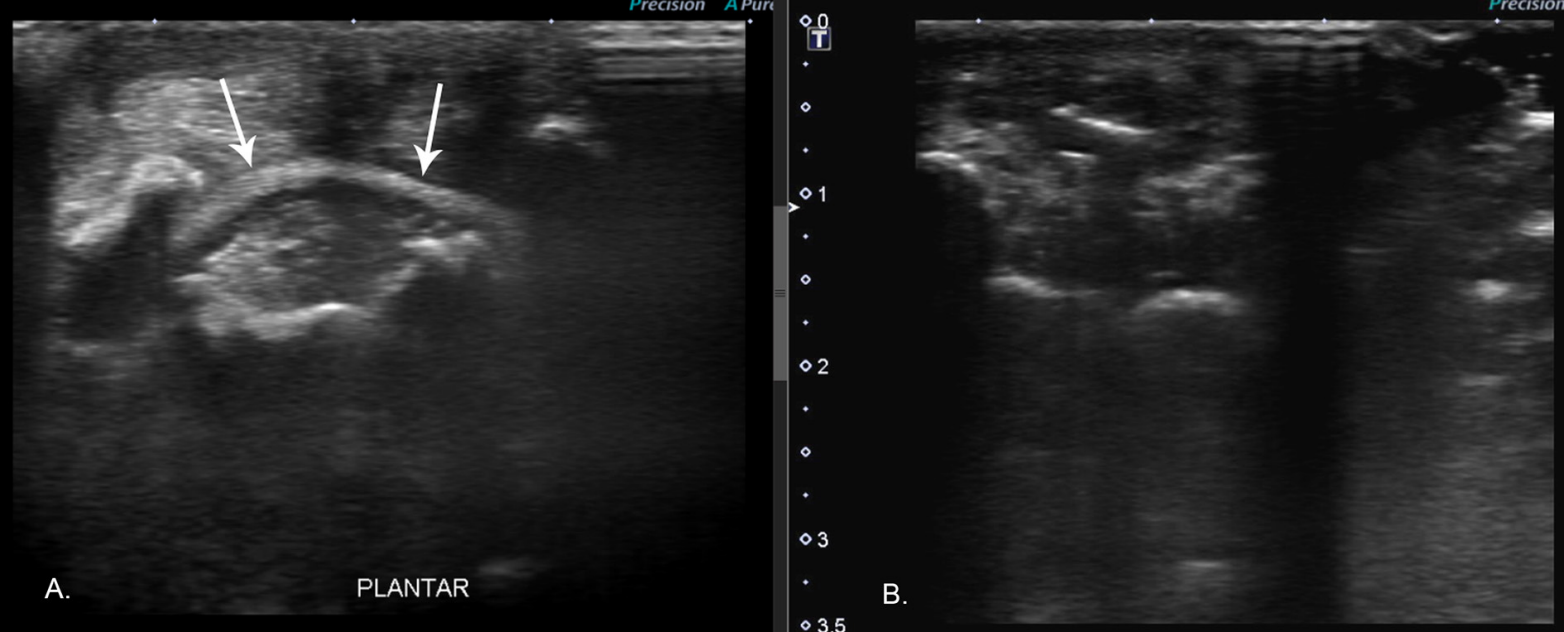
**(A)** Preoperative ultrasound image showed a horizontally oriented hyperechoic band (arrows) along the plantar aspect of the limb deep to the flexor tendons and superficial to the metatarsal bones. **(B)** Intraoperative ultrasound along the same imaging plane demonstrates the absence of foreign material.

Three-view thoracic radiographs (*Ysio Max, Siemens, Erlangen Germany*) were assessed for anesthetic co-morbidities and were concluded to be unremarkable. A brief ultrasound of the metatarsal swelling was done (*Toshiba Aplio 500 Machine*, 18–7 MHz linear probe), which showed a hyperechoic linear structure along the plantar soft tissues and dorsally between the metatarsal bones (Figure 2A). The cytology of the draining tracts was performed using fine needle aspiration, which identified neutrophilic inflammation with no infectious organisms seen.

**Figure 3:**
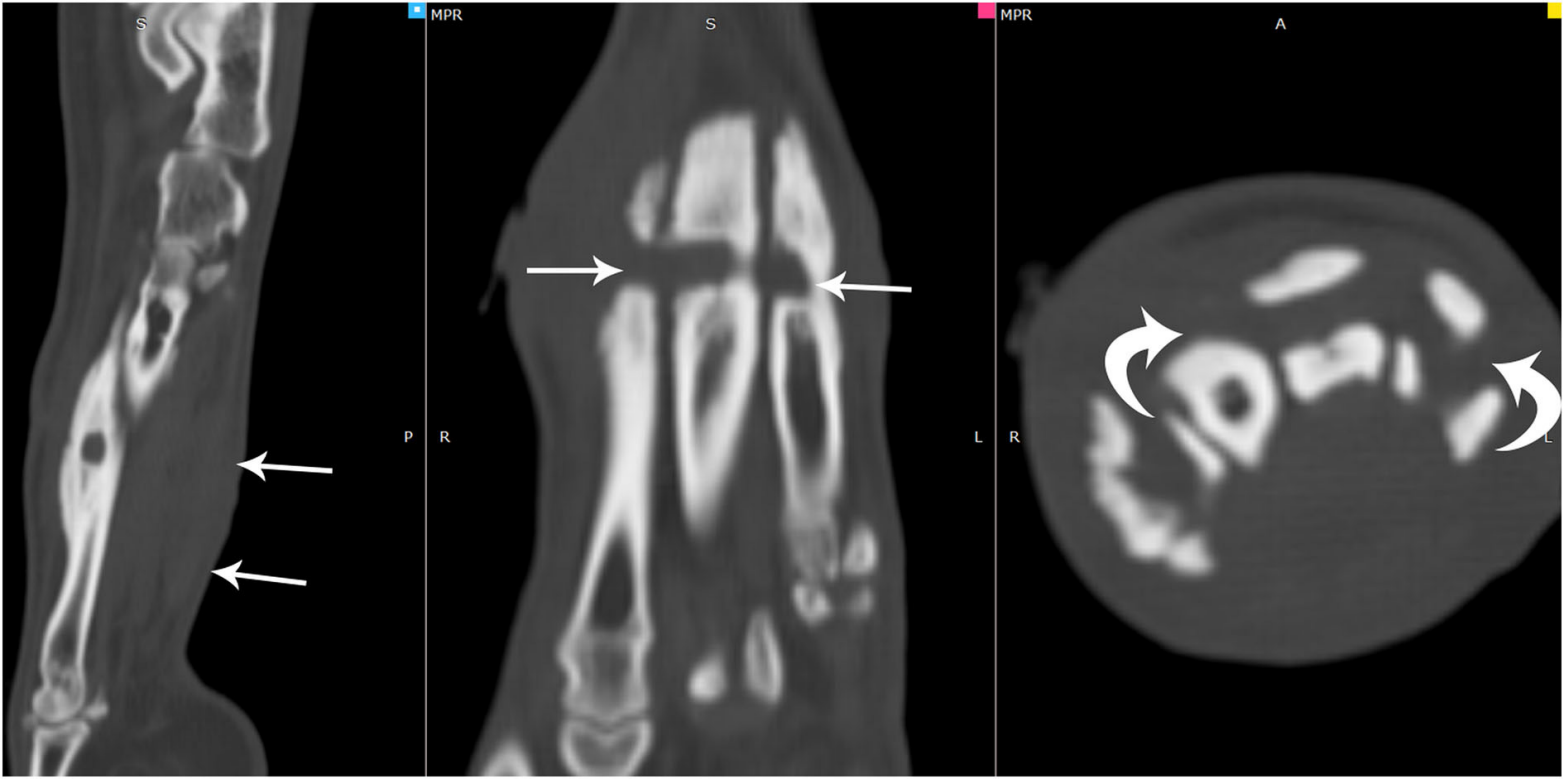
Multiplanar reformatted images of the computed tomographic in a bone window showed soft tissue swelling noted along the plantar aspect of the metatarsal bones (arrows). In this region, there were well-defined smoothly marginated lucencies that appeared to tunnel horizontally through all metatarsal bones (arrows). Adjacent sclerosis and smoothly marginated new bone were noted along the metatarsal bones in this region.

**Figure 4:**
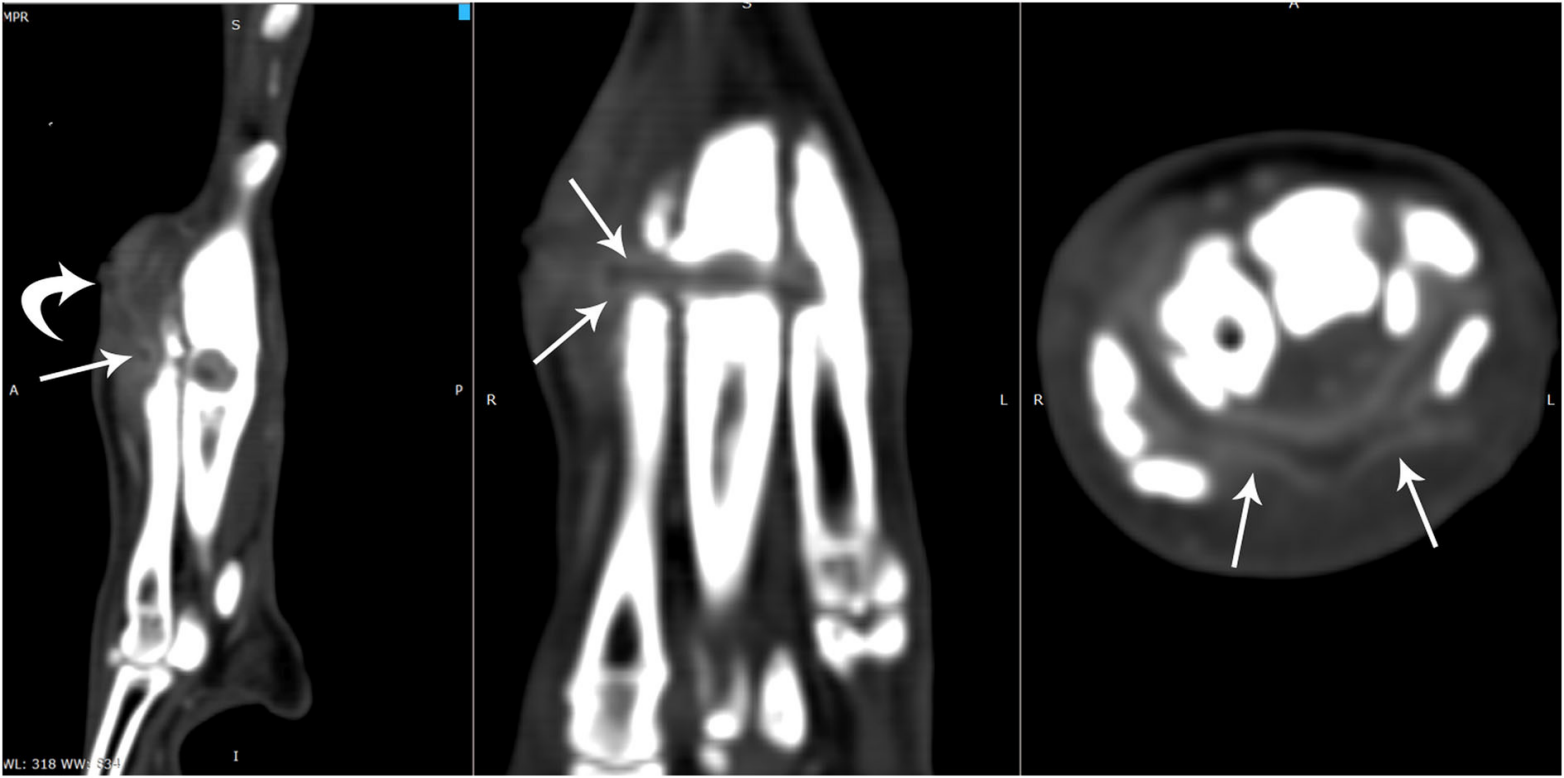
On the post-contrast multiplanar computed tomographic images obtained in a soft tissue window, the non-contrasting tubular structure (arrow) appeared to be adjacent to a soft tissue tract with mild contrast enhancing walls dorsomedial to the 2nd metatarsal bone (curved arrow). There was strong contrast enhancement surrounding a non-contrast enhancing tubular structure (arrows) which circumferentially coursed through the areas of bone lucencies and soft tissues and continued along the plantar region between the interosseous muscles and the flexor tendons. The flexor tendons appeared indistinct in this region.

A CT (*CT; GE Lightspeed VCT 64 Slice*, GE^®^, USA) study was performed on the distal rear limbs to further investigate potential causes for the patient's persistent draining tracts and intermittent lameness. Images were acquired in soft tissue and bone algorithms using *(120 KV 200 MA*) the patient in dorsal recumbency. Multiplanar reconstruction was done using a DICOM viewer tool (RadiAnt Poznan, Poland; https://www.radiantviewer.com/) (Figure 3). Post-contrast images were acquired after intravenous administration of 58 ml of Iopamidol (Isovue 370) *via* an intravenous catheter (Figure 4).

It was concluded that the horizontal circumferential non-contrast enhancing tubular structure surrounding the right metatarsal bones with tunneling into the 2nd through 5th metatarsal bones with focal reabsorption of the 4th metatarsal bone was most consistent with a chronic strangulating foreign body. The adjacent soft tissue swelling and subtle undulate margins at the level of the boney lysis were consistent with the focal inflammation, draining tracts, and the embedded foreign body. The indistinct appearance and thickening of the plantar soft tissues likely represented tendinous injury to the flexor tendons.

Exploratory surgery at AUCVM was done the following day. A medial surgical approach was made to the mid-second metatarsal in the location of the two medial sinus draining tracts based on CT evaluation of the most superficial location of the foreign material. The soft tissues of the metatarsus were dissected down to the second metatarsal bone, and a round lytic area was identified, as seen on CT. Suction was applied to this area, and a black rubber band was easily removed using suction. Intra-operative ultrasound was performed (Figure 2B), using the same ultrasound machine with the 18–7 MHz linear probe, and no further foreign material was identified. The tissues were explored surgically toward the other draining sinuses, but no additional foreign material was found. The surgical wound was copiously lavaged, and the subcutaneous and cutaneous layers were closed with 3–0 Monocryl and Ethilon suture in simple continuous and cruciate patterns, respectively.

Recovery from anesthesia was uneventful and the patient was discharged a day after surgery. During hospitalization, the patient developed diarrhea so the carprofen was stopped. He was sent home with acetaminophen and codeine (300/30 mg tablet, 1 tab PO Q 12 h), as well as trazodone (150 mg, 1 tab PO Q 8–12 h). The clindamycin was continued further for an additional 2 weeks. The carprofen (100 mg, ½ tab PO Q 12 h) was directed to not be continued unless the patient had passed normal stools for 24 h. The patient was discharged with a right distal limb bandage to be changed in 2–3 days and continued until the discharge resolved.

## Discussion

Rubber band syndrome presents similarly in humans and animals. The presence of a circumferential, linear wound or scar, chronic draining tracts or purulent discharge from wounds, edema, pain, lameness, and reduced range of motion should raise suspicions of a strangulating foreign body, especially if occurring on the distal limbs. While osteomyelitis can result concurrently, any of the aforementioned signs should raise suspicions of a foreign body and further exploration of the wound may be warranted. Misdiagnosis of rubber band syndrome as primary osteomyelitis has occurred in human medicine and should be avoided if close attention is paid to clinical presentation and radiographic evidence (1).

Radiographic features of rubber band syndrome, as seen in the RDVM radiographs, include a soft tissue constriction sign (2). The constriction sign can be identified as sharp, constricting indentations of the soft tissue at the level of the foreign body, indicating strangulation and compression of soft tissues. This sign was noted in 78.57% of human case studies with rubber band syndrome (7). Focal osteolytic changes consistent with ischemic necrosis in a tubular or tunneling pattern, boney remodeling, regional periostitis, poor progression of lysis or periosteal reaction on repeat radiographs, and soft tissue swelling were noted in the referral radiographs. These findings are consistent with previous reports of rubber band syndrome in distal limbs of humans and animals (1, 3, 5, 7, 8, 10, 14, 15, 18, 19).

Ultrasound was used before and during surgery to assist in the identification of foreign material and to ensure complete foreign body removal. Two articles described success in visualizing the echogenic foreign body within the lesion using high-resolution ultrasound (3, 19). In a similar case involving the metacarpals of a dog, ultrasound was not diagnostic of the foreign material possibly due to the encircling of the band within the metacarpals (9). In our case, the band had migrated into the metatarsals, but ultrasonography was still able to detect the foreign body when not surrounded by bone. The hyperechoic band noted on ultrasound facilitated the diagnosis of rubber band syndrome. If an elastic rubber band or hair band is suspected, a hyperechoic horizontal band may be seen on ultrasound and can aid in diagnosis.

There is only one case report, to the author's knowledge, in a 3-year-old Chihuahua, involving a circumferential cervical rubber band foreign body, that describes CT characteristics of rubber band syndrome in veterinary medicine (13). A circumferential, linear foreign body was seen in both studies; however, the foreign body in this case was not as markedly hyperattenuating on the pre-contrast images. Both cases did have a post-contrast enhancing rim surrounding the foreign body and the draining tracts. Tunneling pattern osteolysis, sclerosis, and callus formation on dorsolateral to dorsomedial aspects of affected bones were found in our CT study. As corroborated by the previous case report, minimal to mild periosteal reaction was seen surrounding affected bones, which may be a more common characteristic of rubber band syndrome (without secondary osteomyelitis) and could aid in reducing suspicion for aggressive etiologies (13).

## Conclusion

This report documents the diagnosis, treatment, radiographic, ultrasonographic, and CT characteristics of rubber band syndrome in the distal right hind limb of a dog. Additionally, this is one of only two reports (13) that detail CT characteristics of this syndrome in veterinary medicine and adds to the literature by detailing rubber band syndrome in animals. This case highlights the importance of including rubber band syndrome as a differential in any patient that presents with lameness, non-healing wounds or healed wounds that develop draining tracts, circumferential scars, and edema or swelling distal to a wound or scar. Radiographic evidence of a soft tissue constriction sign, mild periosteal reaction, boney lysis or remodeling, tunneling osteolysis, and mild to no progression on recheck radiographs should further raise suspicions for rubber band syndrome. Ultrasound may aid in the confirmation of annular constricting foreign material and can be used to confirm surgical excision. CT can be used to further identify the presence of foreign material and is important for surgical planning.

## Data availability statement

The original contributions presented in the study are included in the article/supplementary material, further inquiries can be directed to the corresponding author.

## Ethics statement

Ethical review and approval was not required for the animal study because this is a client owned animal that presented to the AUCVM Hospital for treatment of an intermittent lameness, draining tract, and bone lysis. Written informed consent was obtained from the owners for the participation of their animals in this study.

## Author contributions

HW, KP, MH, and KF identified the case to be included in the study, performed the image evaluation, and drafted the initial manuscript. All authors contributed to the manuscript's final revision and approved the submitted manuscript.

## Conflict of interest

The authors declare that the research was conducted in the absence of any commercial or financial relationships that could be construed as a potential conflict of interest.

## Publisher's note

All claims expressed in this article are solely those of the authors and do not necessarily represent those of their affiliated organizations, or those of the publisher, the editors and the reviewers. Any product that may be evaluated in this article, or claim that may be made by its manufacturer, is not guaranteed or endorsed by the publisher.

## References

[1] Agarwal A Agarwal S. Retained foreign body masquerading as chronic osteomyelitis: a series of 3 cases and literature review. J Clin Orthop Trauma. (2019) 10:816–21. 10.1016/j.jcot.2018.05.018 31316264 PMC6611954

[2] Aggarwal AN Kini SG Arora A Singh AP Gupta S Gulati D. Rubber band syndrome-high accuracy of clinical diagnosis. J Pediatric Orthop. (2010) 30:e1–4. 10.1097/BPO.0b013e3181e0cb8a 20864842

[3] Baddula AR Yalamanchili RK Vuthpala VM. A case report of osteomyelitis of lower end of tibia and fibula as a complication of elastic rubber band syndrome (dhaga syndrome). J Orthop Case Rep. (2021) 11:56. 10.13107/jocr.2021.v11.i04.2152 34327167 PMC8310633

[4] Boonwittaya N Kaewmanee S. Rubber band syndrome in a dyspneic dog. Thai J Vet Med. (2020) 49:377–83. Available online at: https://he01.tci-thaijo.org/index.php/tjvm/article/view/240421

[5] Brisson BA Théoret MC. Osteolysis of the radius and ulna induced by a circumferential foreign body in a cat. J Am Vet Med Assoc. (2008) 233:1117–20. 10.2460/javma.233.7.1117 18828723

[6] De Armond C Niimura del Barrio MC Rosati T McAllister H Ryan J. Tracheal constriction in a growing dog. Vet Record Case Rep. (2017) 5:e000399. 10.1136/vetreccr-2016-000399

[7] John R Khurana A Raj NG Aggarwal P Kanojia R Chayapathi V. The 'forgotten rubber band'syndrome—a systematic review of a uniquely 'desi'complication with a case illustration. J Clin Orthop Trauma. (2019) 10:822–7. 10.1016/j.jcot.2018.04.014 31316265 PMC6611951

[8] Kurup JKN Shah HH. A rare case of rubber band syndrome of wrist with distal radius and ulna fracture. J Orthop. (2020) 20:60–2. 10.1016/j.jor.2020.01.010 32042231 PMC7000557

[9] Meyers RC Diener MK Cohen EB. What is your diagnosis? J Am Vet Med Assoc. (2022) 260, 1452–3. 10.2460/javma.20.11.0632 35298401

[10] Modransky PD Welker B Carrig CB Sierra C. Osteomyelitis and ankylosis of the pastern joint caused by an elastic hair band. Equine Pract. (1991) 13, 20–4.

[11] Sadler VM Wisner ER. What is your diagnosis? Circumferential foreign body within the soft tissues of the neck and pronounced tracheal compression. J Am Vet Med Assoc. (2000) 216:1723–4. 10.2460/javma.2000.216.1723 10844961

[12] Sosnouski D Chapin RW Thacker PG Walton ZJ Mooney JF. MRI diagnosis of rubber band constriction syndrome. Radiol Case Rep. (2020) 15:999–1001. 10.1016/j.radcr.2020.04.039 32426084 PMC7226657

[13] Stelmach D Sharma A Rosselli D Schmiedt C. Circumferential cervical rubber band foreign body diagnosis in a dog using computed tomography. Can Vet J. (2014) 55:961. 25320384 PMC4187375

[14] Vigneshwaran S Inbaraj C Monica G Rama T Chandrasekaran D. Rubber band syndrome in a pup: an unusual case report. Pharma Innovation J. (2021) 10:231–2.

[15] Watson E Niestat L. Osseous lesions in the distal extremities of dogs with strangulating hair mats. Vet Radiol Ultrasound. (2021) 62:37–43. 10.1111/vru.12924 33184951

[16] Werner-Gibbings K Ischia L Khoma O Tang R. Forgotten elastic band as an unusual cause of limb ulceration: case report and review of the literature. Case Rep Med. (2019) 2019:6195967. 10.1155/2019/6195967 31396282 PMC6664543

[17] Westermeyer HD Tobias KM Reel DR. Head and neck swelling due to a circumferential cicatricial scar in a dog. J Am Anim Hosp Assoc. (2009) 45:48–51. 10.5326/0450048 19122065

[18] Yang G Huang Y Ye W Yu H Mei H. A rare case report of acquired rubber band syndrome due to an unnoticed rubber band on a baby's ankle. Transl Pediatr. (2020) 9:66. 10.21037/tp.2020.01.03 32154137 PMC7036637

[19] Ye W Li H Xu L. Case Report Rubber band syndrome in children: a case report and literature review. Int J Clin Exp Med. (2019) 12:7874–7.

